# Evaluating the Impact of Institutional Performance and Government Trust on Farmers’ Subjective Well-Being: A Case of Urban–Rural Welfare Gap Perception and Family Economic Status in Shaanxi, Sichuan and Anhui, China

**DOI:** 10.3390/ijerph20010710

**Published:** 2022-12-30

**Authors:** Xiuling Ding, Qian Lu, Lipeng Li, Apurbo Sarkar, Hua Li

**Affiliations:** 1 College of Economics and Management, Northwest A&F University, Xianyang 712100, China; 2 School of Economics and Management, Ningxia University, Yinchuan 750021, China

**Keywords:** institutional performance, government trust, well-being, welfare gap, farm household, economic status, structural equation modelling, AMOS

## Abstract

In the modern world, fostering comprehensive social sustainability has become one of the major concerns. Interestingly, rural livelihood may significantly comprise the compelling performance evaluations of governmental institutions’ performances. Governmental institutions’ performances in rural areas largely depend on whether they can gain relatively higher trust levels of marginal farmers. However, the critical interaction between these two prospects may foster farmers’ subjective well-being (SWB). Therefore, the study aims to model and test institutional performance, government trust, and farmers’ subjective well-being by utilising a survey of data from 963 farmer households in Shaanxi, Sichuan, and Anhui provinces, China. We have adopted structural equation modelling (SEM) to craft the study’s findings. However, in the literature, political performance is widely quantified by the urban–rural welfare and economic status gap; thus, in the core model, we have incorporated and measured the mediating role of the urban–rural welfare gap and household economic status. The results show that institutional performance, social insurance performance, and ecological livability performance have a significant and positive impact on institutional performance and government trust and eventually derive farmers’ SWB. However, the role of environmental livability performance is more substantial than social insurance performance in quantifying governmental trust and institutional performance. Moreover, it has a significant positive impact on the subjective well-being of farmers, and the effect of policy trust is not substantial. The results of further mediation and moderation effects show that social insurance performance and ecological livability performance can enhance the subjective well-being of farmers through the indirect transmission of institutional trust. In contrast, the mediating impact of policy trust is not significant. For farmers with higher economic status, institutional performance has a more substantial effect on the subjective well-being of farmers with a relatively smaller perception of the urban–rural welfare gap and lower family economic status.

## 1. Introduction

The notion of social sustainability refers to the practices that incorporate a healthy and socially viable present and future and maintain a close relationship with socially significant organisations that assist in achieving those [1,2]. In emerging countries, the social well-being of general households mostly depends on governmental interventions and their relative policy performance [3,4]. Subjective well-being (SWB) is the term used to describe how individuals perceive and assess their life and specific environments and actions [5]. According to the theory, each person has a specific foundation that can change SWB depending on their circumstances, perspectives, and evaluations of their lifestyles in general and particular genres and actions within their livelihoods [6,7]. Researchers (such as De Rito et al. [8], Huang [9] and Sinha et al. [10]) have used this knowledge to understand the human emotions and perspectives of individuals from diverse stages of life, family, communities, and social systems. According to De Neve et al. [11], changes in the prospects of the subjective well-being of general people have wide-ranging effects on any country’s economic, social, and health outcomes. Thus, the existing literature broadly explored a distinct cohesion between SWB and sustainable development goals (SDGs), as SDGs are a broad collection of policy objectives that seek to eradicate global poverty and hunger, deal with climate change and safeguard the ecosystem, and guarantee everyone has the opportunity to decent healthcare, schooling, and inclusivity [12,13,14]. Nowadays, researchers are becoming more interested in studying the impacts of subjective well-being and what it causes and focussing on revealing details about non-material attributes that may affect how they act in the economy. Moreover, social welfare is a desirable human demand which can be impacted by the legitimacy of the government’s governance and reflect the government’s level of reasonable control [15,16]. However, a strong association between subjective well-being and the inclusive development approach may exist as this strategy focused mainly on incorporating rural inhabitants into core growth settings [17,18]; hence, this approach may have a considerable bearing on subjective well-being.

Since the reform and opening up, the material conditions of Chinese residents have been greatly improved by rapid economic growth, and a multi-fold increase in per capita income [19,20], and the demand for happiness has also exceeded that of any time before [21,22]. However, their subjective well-being has not been effectively improved due to material abundance but has fallen into the so-called “happiness trap” [23,24]. Improving farmers’ livelihood and social obligation has become an integral part of social sustainability and is broadly highlighted as the primary obligation of sustainable development [25]. Similarly, improving farmers’ livelihood and social obligation has become an integral part of social sustainability and is broadly highlighted as the primary obligation of sustainable development [26,27]. There are more than 500 million farmers in China, and the unbalanced development between urban and rural areas is reflected in material aspects and subjective well-being [28,29]. The government, academia, and development organisations widely endorse farmers’ health, safety, and social well-being. Therefore, how to adapt to the pursuit of a better life for farmers and effectively improve their subjective well-being has become a major practical problem that governments at all levels need to solve urgently.

Aligning with this notion, the 19th National Congress of the Communist Party of China raised the improvement of people’s livelihood as a strategic touchstone for testing the nature of a political party and a regime and put forward the reform and development goals of “enhancing people’s sense of fulfilment, happiness, and security, more secure, and more sustainable” [30]. The Fourth Plenary Session of the 19 th CPC Central Committee further proposed to “adhere to and improve the people’s livelihood security system that coordinates urban and rural areas, improve the basic public service system, and focus on strengthening the construction of people’s livelihood that is inclusive and basic” [31]. As for the effect of the livelihood security system on the subjective well-being of farmers, scholars mainly study whether the livelihood security system is implemented, the type of implementation, and the level of investment, and conclude that “government spending on livelihood security can increase residents’ well-being” and “new rural security system” [32,33,34]. A series of positive conclusions, such as the new rural cooperative medical system, pollution control, and other livelihood security systems, have a stronger positive effect on the subjective well-being of disadvantaged groups in farmers [35].

As the urban–rural development gap and the risks faced by farmers’ survival and development are expanding, the role of the government’s livelihood security system on farmers’ subjective well-being has gradually become popular. Existing literature argued that the subjective well-being of farmers is related to personal characteristics (gender, age, marriage, etc.) [36,37], family characteristics (income, consumption) [38,39], social capital (relationship network, social trust) [40,41], and macroeconomic factors (economic growth, income gap, unemployment rate) [42,43]. In a study of Vietnamese fish farmers, Duc [44] found that the subjective well-being of the farmers largely depends on how the government deals with the equal growth strategy between urban and rural regions. Nadeem et al. [34] outlined that SWB can be effective when targeted policy directions and economic prospects have been satisfied. They also stated that a higher level of economic growth can lead farmers to ensure household investment rise and a relatively higher level of satisfaction. Liu and Cheng [37] identified a sense of equity regarding urban–rural and regional differences that primarily impact farmers’ SWB. In a study of Iranian rice farmers, Mohammadrezaei et al. [45] outlined that farmers’ perception of economic, social, and environmental well-being significantly explained their subjective well-being constructs, including happiness and life satisfaction. However, it should be emphasised that although implementing the livelihood guarantee system can directly affect farmers’ production and life in practice, and it must also involve the subjective well-being of farmers through the system performance subjectively formed by farmers [34,46]. It is one of the most direct influencing factors of farmers’ subjective well-being. From the micro perspectives of comprehensive rural development strategy, how to adapt to the pursuit of a better life for rural farmers and effectively improve their subjective well-being has become a major practical problem that the Chinese government urgently needs to solve.

Although the existing research (such as Bhuiyan and Ivlevs [47], Wijayanto et al. [48], and Liang and Zhu [49]) have studied the implementation of the people’s livelihood security system and farmers’ subjective well-being, they have paid less attention to measure the role of institutional performance and farmers’ trust on government for fostering farmer’s subjective well-being. Moreover, the fundamental interactions between these two critical variables have not been evaluated integrated by any studies. On the other hand, the existing studies (such as Chen [50], Liu and Cheng [37], Hu et al. [36] and Tang et al. [31]) have separately assessed the impacts of urban–rural welfare gap perception and family economic status regarding farmers’ subjective well-being. Thus, this research first constructs a theoretical model including the perception of the urban–rural welfare gap, institutional performance of household economic status, government trust, and farmers’ subjective well-being, and then constructs a structural equation based on the survey data of farmers in Shaanxi, Sichuan and Anhui, China. To the best of our knowledge, the study will be one of the first attempts to integrate institutional performance, government trust, and farmers’ subjective well-being in an integrated framework. Moreover, the analysis incorporates the mediating role of urban–rural welfare gaps and households’ economic status towards fostering farmers’ SWB, which will be another major innovation of the study. The empirical analysis of the model can not only help to reveal the formation mechanism of farmers’ subjective well-being but also provide a decision-making reference for the government to improve governance performance in rural areas.

The rest of the study is as follows. The Section 2 represents the theoretical outline and hypothesis development. The Section 3 portrays materials and methods used for crafting the study’s outcomes. At the same time, the study outlines its results and findings in Section 4 and the discussion in Section 5. Section 6 is dedicated to presenting the conclusions and policy direction based on the results and discussion presented in the study.

## 2. Theoretical Analysis and Hypothesis

**Figure 1:**
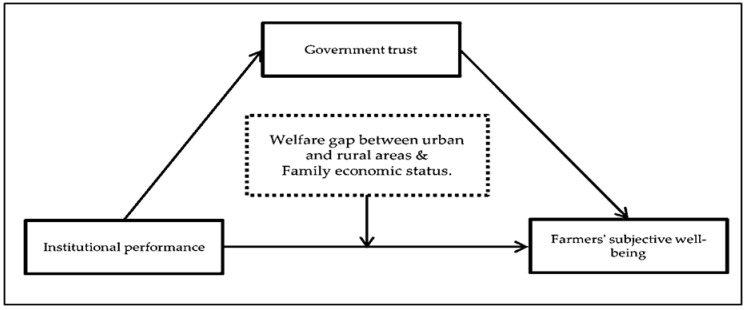
Proposed framework for the study.

The study takes the social insurance system and the ecological livability system as the objective carrier of system performance, combines rural people’s livelihood security system practice, and analyses the relationship between system performance, government trust, and farmers’ subjective well-being to portray the theoretical background. Figure 1 illustrates the theoretical framework of the study:

### 2.1. Institutional Performance and Farmers’ Subjective Well-Being

The inter-relationship between governmental institutions and their performance has long been considered the prime indicator for fostering governmental fiscal, social and organisational support. According to Ott [51], governmental and institutional performance is encouraged mainly by the effectiveness of legislation development and execution and the legitimacy of the government’s adherence to those legislations. However, rural farmers’ livelihood and agro-production are predominantly derived from the optimum performance evaluations of specific legislative considerations. Exiting studies (such as Norman and Kebe [52], Jacobi [53], and Pritchard [54]) outlined that the production and livelihood of farmers are directly subject to government policy direction, which makes individual, institutional performance evaluation closely related to their subjective well-being. According to Boin et al. [55], farmers in a developing country are considered one of the most vulnerable communities and highly depend on governmental institutions. Thereby, their performance mainly depends on how the institutions perform.

Moreover, several other institutional interventions also can provide direct and indirect impacts to rectify the livelihood and well-being of farmers. Things such as endowment insurance can ensure that farmers have a stable source of income when they are old and reduce their worries [56]. Seemingly, medical insurance can ensure that they will not become impoverished due to illness, so they can rely on it [57]. Comprehensive rural infrastructure, facility construction, and environmental pollution control provide a more beautiful and livable environment and make it look forward to the life of farmers [58]. Therefore, improving the institutional performance of social insurance and ecological livability may help farmers to enhance their sense of security, obtain better development opportunities, and significantly improve their subjective well-being. Based on this, the study put forward hypothesis one:

**Hypothesis** **1** **(H1).** *Institutional performance has a significant positive impact on the subjective well-being of farmers*.

### 2.2. Government Trust and Farmers’ Subjective Well-Being

Maximising perceived well-being has been considered a prime aim of governmental and development organisations [59]. Existing studies (such as Balezentis et al. [60] and May et al. [61]) outlined an attitude of believing that behaviour or the surrounding environment conforms to expectations. However, residents’ trust in the government indicates the extent to which they believe it can operate satisfactorily. If the government is trusted or the residents have a positive evaluation of the government, then this sense of trust will become the interaction between residents and the government [62,63]. This eventually enhances residents’ self-identity and dependence on themselves or the surrounding environment and significantly increases their subjective well-being [64,65].

As an indispensable management agency in rural society, the government has enormous power and resources to affect the production and life of farmers, and the belief in state power also makes farmers generally rely on the government [66]. Therefore, when farmers trust the government, they can shape positive values to form stable and optimistic expectations for the future, which significantly increases the subjective well-being of farmers. Conversely, if the government’s trust is continuously lacking, farmers will reduce their recognition and dependence on the outside world, which will seriously shake the foundation of their subjective well-being [13,36]. Due to this, this article proposes hypothesis H2.

**Hypothesis** **2** **(H2).** *Government trust has a significant positive impact on farmers’ subjective well-being*.

### 2.3. The Mediating Effect of Government Trust between Institutional Performance and Farmers’ Subjective Well-Being

Institutional performance can improve farmers’ subjective well-being through the indirect transmission of government trust [67]. This is because, on the one hand, government trust is the confidence of social members in the government, which is based on the results produced by the operation of the government or political system that are consistent with their expectations [68,69]. On the other hand, government trust is the most stable factor affecting farmers’ subjective well-being, enhancing their tolerance and sense of belonging and significantly increasing their subjective well-being and happiness [70]. For farmers, the rural livelihood security system and endowment insurance reduce the worries of farmers when they are old, medical insurance guarantees farmers to rely on when they are sick [68], and the construction of beautiful villages and environmental pollution control meet farmers’ expectations for a better life [71]. Further improvement of the performance of the guarantee system will help enhance farmers’ sense of security, obtain better development opportunities, make farmers feel the government’s concern and policy effectiveness, and enhance farmers’ trust in the government [72].

However, the existing literature (such as Zawojska [73], Ambali and Begho [74], and Taylor and Van Grieken [75]) showed a close inter-relationship between institutional performance and farmers’ trust in government. In a study of Indian farmers, Alkon and Urpelainen [76] found that farmers possessed more trust when the government institutions acted more responsibly. According to the value–belief–norms theory, farmers will have better perceptions regarding governmental policies when they are benefited from perceived social values from the institutions such as agricultural extension, training facilitators, risk-taking networks, and other financial institutions [77]. Therefore, the study hypothesised (H3) as “the institutional performance may not only directly affect the subjective well-being of farmers but have indirect transmission of government trust.

**Hypothesis** **3** **(H3).** *Government trust plays a positive mediating role in institutional performance and farmers’ subjective well-being*.

### 2.4. Moderating Effects of Perception of Urban–Rural Welfare Gap and Household Economic Status on Institutional Performance and Farmers’ Subjective Well-Being

Although the Chinese government has formulated a series of livelihood security systems for rural farmers to improve the rural environment and improve the living standards of farmers [78], the objective development gap between urban and rural areas cannot be eliminated in a short time by the government’s efforts [79]. There is still a significant gap in objective welfare between urban and rural areas, which makes farmers form an empirical cognition of the social welfare gap between urban and rural residents in their daily production and life, which is called the perception of the urban–rural welfare gap in this section [80,81]. In practice, the improvement of institutional performance does help to enhance farmers’ sense of security, obtain better development opportunities, and improve farmers’ subjective well-being [82]. Seemingly, with the development of information and communication technology, rural farmers are becoming more aware of the considerable welfare gap between urban and rural areas and the weakening of the positive effect of institutional performance on farmers’ subjective well-being [83,84].

Family economic status is farmers’ perception of their social class status, often formed by comparing farmers with surrounding groups [85]. This part specifically refers to the subjective economic status of the family created by comparing farmers and other people in the village [86]. The government’s livelihood security system is universal and provides little help to each farmer [87], so its marginal contribution to the welfare improvement of farmers with higher family economic status in the village is negligible [88]. Farmers with lower economic status contribute more to the advancement of welfare and can significantly improve their security level [89], such as medical insurance to ensure that farmers will not become impoverished due to illness or other natural disasters [90]. Therefore, improving institutional performance may substantially affect the subjective well-being of farmers with lower economic status.

**Hypothesis** **4** **(H4).** *Perception of urban–rural welfare gap and household economic status negatively modifies “institutional performance and farmers’ subjective well-being”.*

## 3. Materials and Methods

The study utilised structural equation modelling (SEM) for crafting its outcomes. The SEM is one of the widely used tools by the existing literature within the agriculture domain (such as Ma [91], Sarkar et al. [92], and Biswas and Sarkar [93]) as it can quantify relatively robust outcomes with limited data. Moreover, SEM does not require strict data normality to provide comprehensive outcomes of the latent and observed variables [94]. We used the AMOS21.0 statistical software (IMB, New York City, NY, USA, https://www.ibm.com/products/structural-equation-modeling-sem, (accessed on 11 November 2022)) for performing the SEM estimation and the robust maximum likelihood method. The reason for choosing AMOS is that it delivers an appropriate visual platform, and the software interface is well-designed for new learners. In addition, it provides support for the investigation and hypotheses by expanding basic multidimensional analytic procedures, such as regression, factor analysis, correlation, and analysis of variance [95]. AMOS has more significant superiority than typical multivariate analytical methods like LISREL and other Partial Least Square (PLS) base software like Smart PlS, as it allows one to develop psychological and behavioural frameworks more effectively to depict complicated interactions [92,96]. However, even AMOS has some limitations regarding assessing the proper model fit and having a constraint on the normal distribution [97]; according to the study, AMOS has been chosen.

### 3.1. Data Sources

The data used in the study are from a questionnaire survey of farmers. The research group comprises teachers and postgraduate students, with a total of 12 members. Before the formal investigation, the experts of the research group conducted unified training on questionnaires and research background, including the content of the questionnaires, research methods, and sampling methods, and conducted pre-investigation in Baota District, Yan’an City, and revised and improved the questionnaires. The research team conducted formal farmer surveys in Shaanxi, Sichuan, and Anhui provinces from July to August 2020. Based on considering the economic development of the county and the feasibility of the investigation, ten counties are determined within three provinces, and then from each county (city) randomly selects 3–4 townships. We randomly choose 2–4 villages from each township, and each village selects 10–15 household farmers. The survey limited the interview objects to farmers over 18 with complete cognitive ability and determined it by random interviews.

The survey method was conducted in the form of face-to-face questionnaire interviews, which mainly contained the characteristics of household heads, family characteristics, system performance, government trust, and subjective well-being. In addition, because the reliability of the questionnaire is proportional to the number of points measured by the scale and the fact that it is difficult for ordinary farmers to identify scales above five, the overall Likert 5-level scale is used for measurement. In the questionnaire survey process, each questionnaire is reviewed in time to ensure the completeness and accuracy of the questionnaire data. One thousand twenty interviews were taken during the survey, and 963 valid questionnaires were obtained after excluding the large-scale blanks and missing fundamental data. Which comprehensively met the sample size requirements of the structural equation model as suggested by Wong [98] and Sarkar et al. [99]. The details of the research area and sample farmers are shown in Table 1.

### 3.2. Variable Selection

Based on the literature review, this study selected government performance, institutional trust, and farmers’ subjective well-being as fundamental variables. The variables and objects are described in detail below.

#### 3.2.1. Subjective Well-Being 

Subjective well-being is usually regarded as a value description of people’s mental state, subjective meaning, and life satisfaction [100]. It not only comes from the quality of life but also from abstract psychology and perceived trust [76]. Therefore, referring to the Asian Democracy Dynamics Survey [101], the World Values Survey [102], and the China Comprehensive Social Survey [103], we set up “In general, is your family happy (A1)? Are you satisfied with your current life (A2)?, How is the family’s future (A3)?. However, for comprising the input, we have adopted five Likert scale tactics where 1, 2, 3, 4, and 5 represent very poor, poor, average, good, and very good, respectively.

#### 3.2.2. Institutional Performance

Organisational performance reflects system dynamics and is quantified by the satisfaction level of farmers with the services provided by government institutions [68,104], and 1, 2, 3, 4, and 5 indicate very dissatisfied, less satisfied, average, relatively satisfied, and very satisfied, respectively. Due to this, this article takes satisfaction as the evaluation standard of the performance of various livelihood security systems and asks farmers about the new rural endowment insurance system (A4), the new rural cooperative medical care system (A5), the beautiful rural construction system (A6), the rural pollution and the degree of satisfaction with the governance system (A7).

#### 3.2.3. Government Trust

Since the trust points are different, the measurement of government trust is also different. This study perationaliz government trust from two aspects: (i) institutional and (ii) policy trust. The grass-roots government is the specific implementer of the policy and constitutes the most intuitive and fundamental part of government trust. Drawing on the measurement methods of He et al. [105] and de Vries et al. [106], Howell and Howell [107], the institutional trust is perationalized as farmers’ trust in county-level governments (A8). The trust level of the three-level perationali of township government (A9) and the village committee (A10). Considering that policy trust is an abstract evaluation of policy attributes by farmers from the experience level [108], policy trust is perationalized into policy scientific trust (A11), policy adaptability trust (A12), and policy efficacy trust (A13). Three questions. 1, 2, 3, 4, and 5, represent very distrust, less trust, average, more trust, and very trust, respectively.

#### 3.2.4. Perception of Urban–Rural Welfare Gap and Household Economic Status

To effectively measure the perception of the urban–rural welfare gap, set the question “How do you feel about the social welfare gap between farmers and urban residents?” and 1, 2, 3, 4, and 5 represent very small, small, medium, large, and largest, respectively. To effectively measure the family’s economic status, set the question “Which level is your family’s income in the village?” and 1, 2, 3, 4, and 5 represent the worst, poor, medium, better, and best, respectively.

### 3.3. Model Construction

This article mainly studies the relationship between latent variables such as institutional performance, government trust, and farmers’ subjective well-being. In general, latent variables are challenging to obtain through direct measurement, and it is also difficult to explain the relationship between latent variables. Structural equations can better solve the above problems, where structural equations describe the relationship between unobservable latent variables, while measurement equations describe the relationship between latent variables and their observed variables. Given this, build the SEM model:

Measurement model:

(1) $$X=\Lambda_{y}\eta +\varepsilon$$

(2) $$Y=\Lambda_{x}\eta +\delta$$

Structural model:

(3) η=Bη+Γξ+ζ

In the above formula, η is the endogenous latent variable, ξ is the exogenous latent variable, B is the coefficient matrix of the endogenous latent variable, which is the coefficient matrix of Γ the exogenous latent variable, ζ is the unexplained part, and X is the observation of the exogenous latent variable. Whereas Y the observed variable of the endogenous latent variable, $\Lambda_{x}$ is the correlation coefficient matrix of the exogenous latent variable and its observed variable, which is the correlation coefficient matrix $\Lambda_{y}$ of the endogenous latent variable and its observed variable, and ε δ represents the residual item. In the structural equation of this paper, the endogenous latent variables η are government trust and farmers’ subjective well-being, and the exogenous latent variables ξ are institutional performance.

## 4. Empirical Results and Analysis

### 4.1. Descriptive Statistics

Table 2 summarises the essential characteristics of the sample farmers. Regarding the age of household heads, 12.77% of the households were 45 years old and below, 42.89% were 45–60 years old, and 44.34% were 60 years old and above. Regarding the education level of household heads, primary school and below accounted for 65.11%, junior high school cultural farmers accounted for 28.97%, and high school and above cultural farmers accounted for only 5.92%. Regarding family size, 29.39% of the households had two or fewer members, 66.35% had three to five members, and only 4.26% had six or more members. Regarding the degree of part-time jobs, 60.64% of farmers with high part-time jobs accounted for 39.36% of those with low part-time jobs. Regarding per capita household income, 24.30% of households were 10,000 yuan. Below, 31.57% were 10,000 to 20,000, and 44.13% were 20,000 or more. It can be seen from the above that the characteristics of the sample farmers are consistent with the overall situation of the farmers in the study area and are highly representative.

### 4.2. Reliability and Validity Test

Reliability refers to the measurement results’ degree of consistency or stability [109]. In Table 3, each latent variable’s composite reliability (CR) is above 0.7, indicating that the measurement model has high internal consistency, as recommended by Bagozzi and Yi [110]. Cronbach α is close to 0.7, or above 0.7 (Cronbach *α* > 0.7 is high reliability), indicating that each latent variable has relatively reliable reliability [111]. However, validity refers to how effective measurement is. The scale of this study is based on existing literature, so it has good content and face validity. Meanwhile, the KMO values of the latent variables were all greater than 0.7, and the *χ*^2^ values of the Bartlett sphere test were all significant at the 0.000 level, indicating that factor analysis was suitable (*p* ≤ 0.05), as recommended by Amadu et al. [112]. In Table 3, the standardised load of latent variables and the average variance extraction value (AVE) are all greater than 0.5, and the square root of AVE is greater than the correlation coefficient between it and the latent variable. This indicates the degree of explanation of each item on the corresponding latent variable, and the convergence effect of each latent variable outlined acceptable discriminant and discriminant validity [110,111]. The scale has good reliability and validity and is reasonably fit for performing a structural equation model. Overall, the five factors of farmers’ subjective well-being, social insurance performance and ecological livability performance (belonging to institutional performance), institutional trust, and policy trust (belonging to government trust) are obtained.

### 4.3. Analysis of the Fitting Effect of The Structural Equation Model

Minimum Discrepancy Function by Degrees of Freedom divided (CMIN/DF), Goodness of Fit Index (GFI), Adjusted Goodness of Fit Index (AGFI), Root Mean Square Residual (RMR), and Root Mean Square Error of Approximation (RMSEA) were selected as the absolute fitting evaluation indicators to evaluate the fitting effect of the structural equation. In comparison, Normed Fit Index (NFI), Relative Fit Index (RFI), Incremental Fit Index (IFI), Tucker-Lewis Index (TLI), and comparative fit index (CFI) were selected as the relative fitting index evaluation indicators. Seemingly, P-Ratio and P-NFI were set for streamlined adaptation metrics, and Parsimony Comparative Fix Index (PCFI) is used as an evaluation index for model simplification and adaptation. Table 4 depicts that the evaluation indicators, such as absolute fit, close fit, and simplified fit of the structural equation model, have passed the test, indicating that the overall fitting effect of the structural model is good [97,111]. The specific model fit index, evaluation criteria, and results are shown in Table 4.

### 4.4. Direct Path Analysis

According to the running results of AMOS21.0, the path coefficients between the latent variables of the model are sorted out. The specific outcomes and analysis are as follows.

#### 4.4.1. Institutional Performance and Farmers’ Subjective Well-Being

In institutional performance, social insurance performance and ecological livability performance have a significant positive impact on farmers’ subjective well-being at the 5% and 1% significance levels, and the path coefficient of ecological livability performance is greater than that of social insurance performance. This shows that the higher the social insurance performance and ecological livability performance, the higher the subjective well-being of farmers and the effect of environmental livability performance on farmers’ well-being is more vital than that of social insurance performance. Moreover, it indicates that the higher the system performance, the higher the security level of farmers, which helps enhance farmers’ sense of security, obtain better development opportunities and improve their subjective well-being. The effect of ecological livability performance on farmers’ subjective well-being is more robust than that of social insurance performance, which may be closely related to the fact that farmers’ current demand for ecological livability is relatively strong, and the “cost performance” of the social insurance system is declining. Therefore, hypothesis one (H1) is validated.

#### 4.4.2. Government Trust and Farmers’ Subjective Well-Being

Among government trust, institutional trust has a significant positive impact on farmers’ happiness at the 1% level of significance, while policy trust has no significant effect on farmers’ satisfaction. This shows that the higher the institutional trust of farmers, the higher their subjective well-being, and institutional trust has a more substantial effect on farmers’ happiness than policy trust. Therefore, hypothesis two (H2) is verified. As an indispensable management agency in rural society, the government has significant power to influence the production and life of farmers. When farmers trust the government enough, it can enhance their tolerance, sense of belonging, and support in a risky society, making them form a stable and optimistic expectation for the future and increasing their subjective well-being. The effect of institutional trust on farmers’ subjective well-being is more robust than that of policy trust because the carrier of institutional trust is more specific and easier to be the attribution object of farmers’ subjective well-being.

#### 4.4.3. Institutional Performance and Government Trust

In institutional performance, social insurance performance and ecological livability performance have a significant positive impact on institutional trust and policy trust, and the path coefficient of ecological livability performance is greater than that of social insurance performance. The higher the housing performance, the higher the confidence in the government and the role of ecological livability performance is more potent than social insurance performance. Therefore, we can assume that hypothesis three is validated. For farmers, endowment insurance reduces the worries of farmers when they are old, medical insurance guarantees farmers to rely on when they are sick, and the construction of beautiful villages and environmental pollution control give farmers better expectations for life. Therefore, performance improvement can make farmers feel the government’s contribution and policy effectiveness and enhance farmers’ trust in the government. The effect of ecological livability performance on the subjective well-being of farmers is more robust than that of social insurance policy performance, which may be related to the high transparency of the implementation of ecological livability, the straightforward observation of the effect, and the more inclusiveness. Table 5 represents Model Fitting Path Coefficients.

### 4.5. Analysis of the Mediating Effect of Government Trust

Government trust is tested by the Bootstrap method, which has been widely recognised in recent years, and the sampling times are set to 5000 as recommended by Byrne [113]. The nonparametric percentile method with bias correction is selected, and the confidence level of the confidence interval is 90%. The research results show that: in the path “ecological livability performance→institutions trust→farmers’ subjective well-being”, ecological livability performance can have a significant positive impact on farmers’ subjective well-being through institutional trust (coefficient = 0.06, *p* = 0.04, and confidence interval does not contain 0). In the path of “social insurance performance→institutions trust→farmers’ subjective well-being,” ecological livability performance can significantly and positively impact farmers’ subjective well-being through institutional trust (coefficient = 0.06, *p* = 0.04, and the confidence interval do not contain 0). The standard regression coefficients and confidence intervals of the two paths of “ecological livability performance→policy trust→farmers’ subjective well-being” and “social insurance performance→policy trust→farmers’ subjective well-being” failed to pass the mediation effect test. Therefore, hypothesis four (H4) is only partially validated. Table 6 represents the outcomes of mediation test. 

### 4.6. Analysis of the Moderating Effect of Perception of Urban–Rural Welfare Gap and Household Economic Status

With the perception of the urban–rural welfare gap and household economic status as moderator variables, the absolute fit index, relative fit index, and simplified fit index of the multi-group structural equation have all passed the test. This indicates that the multi-group structural equation fits well with the sample data. The specific results and analysis are as follows.

Taking the perception of the urban–rural welfare gap as the moderating variable in the path of the positive impact of ecological livability performance on the subjective well-being of farmers, the perception of the urban–rural welfare gap is more relatively minor (*β* = 0.166, *p* < 0.01) and the urban–rural welfare gap are larger (*β* = 0.097, *p* < 0.10) were significantly affected. In comparison, the path coefficient of the earlier outcomes was greater than that of the latter. In the path of the positive impact of social security performance on the subjective well-being of farmers, the perception of the urban–rural welfare gap was smaller (*β* = 0.1117, *p* < 0.01) has a significant impact, and the perception of the urban–rural welfare gap is substantial (*β* = 0.004, *p* = 0.503). This denotes that it failed the test. Therefore, it can be assumed that hypothesis five is endorsed. Currently, the interaction between urban and rural areas is becoming more and more frequent [108]. The high-quality life of urban residents and a good development environment make disadvantaged farmers more and more aware of the considerable welfare gap between urban and rural areas. The positive effect of perception of the subjective well-being of smaller farmers is robust [114]. Table 7 represents the outcomes of moderating test. 

**Figure 2:**
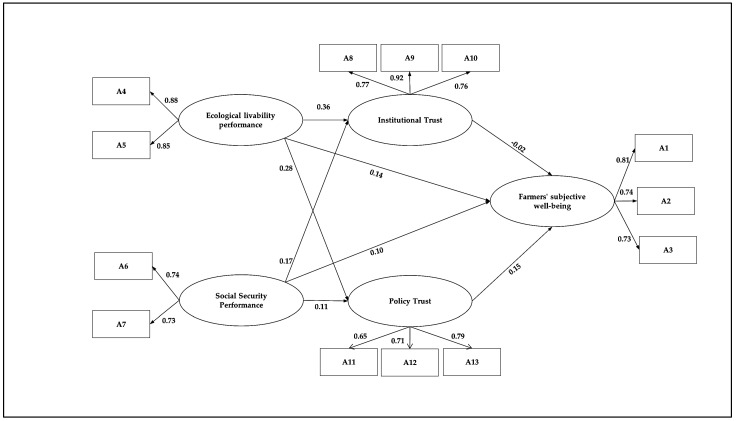
Complete results of SEM frameworks.

Taking the family economic status as the moderating variable, in the path of the positive impact of ecological livability performance on the subjective well-being of farmers, the lower family economic status (*β* = 0.215, *p* < 0.01) has a significant effect, and higher family financial status (*β* = 0.073, *p* = 0.485) failed the test. Likewise, the path coefficient of the former was greater than that of the latter. While, in the path of the positive impact of social insurance performance on farmers’ subjective well-being, the family economic status was lower (*β* = 0.134, *p* < 0.05), and the effects are significant. The family’s higher economic status (*β* = 0.049, *p* = 0.635) fails the test, and the path coefficient of the earlier is greater than that of the latter. Therefore, it can be assumed that hypothesis six is accepted. Compared with farmers with higher family economic status, livelihood security systems such as social insurance and ecological livability can significantly improve the security level of farmers with lower family financial status so that they can have a stable source of income when they are old and will not suffer from illness when they are sick. Therefore, institutional performance substantially affects the subjective well-being of farmers with lower economic status. Figure 2 represents the complete SEM framework and the associated linkage between the key variables.

## 5. Discussion

According to the Food and Agriculture Organization of the United Nations (FAO), the assistance given to agriculture does not provide the outcomes that are wanted for the environment and human wellbeing, but repurposing it may indeed be a game-changer [115]. Governmental bodies and trust in government are interconnected notions that may affect farmers’ behaviour and subjective well-being. Moreover, several governmental, institutional and policy performance matrices may effectively rectify the urban–rural welfare gap and family economic status. The current study results show that: (i) institutional performance, social insurance performance, and ecological livability performance have a significant positive impact on government trust (institution trust, policy trust) and farmers’ subjective well-being. The role of environmental livability performance is relatively more substantial than social insurance performance. Theoretically, subjective well-being can ultimately and reasonably judge the overall satisfaction of farmers’ conditions and emotions, enhance farmers’ living standards [116], improve the social welfare of rural residents [38], adapt to social development needs, and promote the overall development of people [45]. In a study of 15 European nations, Hudson [100] found a similar assumption when respondents are farmers. (ii) In terms of government trust, institutional trust has a significant positive impact on farmers’ subjective well-being, and the mediating effect of policy trust is insignificant. Interestingly, the outcomes differ from the study of Liang et al. [42], which found that policy trust possessed a significant positive interaction. The results of further mediation and moderation effects also show that social insurance and ecological livability performance indirectly enhance farmers’ subjective well-being through institutional trust, and the mediating effect of policy trust is insignificant. (iii) For farmers with higher family economic status, institutional performance significantly impacts the subjective well-being of farmers with more minor perceptions of the urban–rural welfare gap and lower family economic status. The notion is supported by the study of Liu and Cheng [37], Markussen et al. [33] and Agrawal et al. [117].

## 6. Conclusions

In terms of facilitating the farmer’s well-being, accessibility, availability, and acceptability of services are critical factors that signify governmental institutions’ performance and can effectively foster farmers’ trust in governmental policies and supports. However, previous studies have paid minimal attention to exploring institutional performance’s role in fostering farmers’ subjective well-being. Moreover, the distinct impacts of institutional performance on the farmer’s subjective well-being under different prospects of urban–rural welfare gap and family economic status have not been comprehensively explored. In addition, the interaction between institutional performance, government trust and farmers’ subjective well-being has not been fully grasped by the existing literature. Because of this, for the first time, as far as our knowledge, the study evaluates institutional performance, government trust, and farmers’ subjective well-being in an integrated framework and evaluates the comprehensive interaction among those factors. Likewise, the study includes the prospects of the urban–rural welfare gaps and household economic status of farmers into the core analytical framework. Then, based on the survey data of farmers in Shaanxi, Sichuan and Anhui provinces, it establishes a structural process model for empirical analysis, explains its internal logic, and provides a reference for relevant studies and the government’s policy formulation. Based on the findings crafted by the study, the following policy direction has been forwarded.

First, as Chinese farmers have a long history of poor health outcomes, comprehensive strategies are needed to encourage and assist farmers in maintaining better governmental trust, availing a more constructive approach to their well-being, and providing enough confidence to ask for assistance when needed. The government should establish and highlight the improvement of smallholder farmers’ livelihood standards as the core indicator of government performance evaluation and increase the priority of the rural livelihood security system. Moreover, the government should extend financial support for people’s livelihood security systems, such as social insurance and ecological livability, and narrow the objective social welfare gap between urban and rural areas. So farmers relying on the continuously optimised livelihood security system can obtain relatively higher satisfaction and thus enhance their trust in the government. Second, upgrade the performance evaluation system and feedback mechanism of the people’s livelihood security system, strengthen the collection of farmers’ demand information and identify the “shortcomings” of the farmer’s livelihood security system. Third, the government should expand the communication channels for farmers to participate in and discuss several policies and strengthen the information exchange opportunity with target farmers through group meetings, television, short videos, and other forms. The content and latest results of the livelihood security system of model villages should be broadly circulated to improve farmers’ understanding and thus enhance the knowledge spillover impacts and improve farmers’ subjective well-being.

In future research, potential authors can expand the connotation and types of institutional performance, introduce more regulatory variables of farmers’ characteristics and urban–rural gap measurement, and build a more realistic decision-making scenario. Moreover, every region has its characteristics and may be impacted by several control variables. Therefore, the potential studies should explore more regions with diverse farmers’ categories to deeply reveal the effects of institutional performance and government trust on farmers’ subjective well-being. They should extend the model and evaluate the possible direct impacts. On the other hand, the study utilised Structural Equation Modelling (SEM) as a prime analytical tactic with some limitations in ranking variables. Thus, the identified variables can be evaluated, ranked and articulated by other structured evaluating and modelling tactics such as Interpretive Structural Equation Modelling (ISM) and Slacks-based measure (SBM) modelling.

## Figures and Tables

**Table 1:** Research area and sample distribution.

Province	Shaanxi				Sichuan			Anhui		
County	Xixiang	Ziyang	White River	Hanbin	Wang Cang	Tongjiang	Mount Emei	Jinzhai	Qimen	Huangshan
Sample size	81	107	111	81	91	100	103	97	94	98
Proportion (%)	8.41	11.11	11.53	8.41	9.45	10.38	10.70	10.07	9.76	10.18

**Table 2:** Statistics of essential characteristics of sample farmers.

Index	Options	Frequency	Proportion (%)
Age of head of household	45 years old and below	123	12.77
	45–60 years old	413	42.89
	60 years old and above	427	44.34
Education level of the head of the household	Elementary school and below	627	65.11
	junior high school	279	28.97
	High school and above	57	5.92
Family size	Two people or below	283	29.39
	3 to 5 people	639	66.35
	Six people and above	41	4.26
Part-time degree	Low (≤50% of non-agricultural income)	379	39.36
	High (the proportion of non-agricultural income > 50%)	584	60.64
Per capita household income	10,000 yuan and below	234	24.30
	10,000 to 20,000	304	31.57
	more than 20,000	425	44.13

**Table 3:** Reliability and validity test results.

Variable		Item	Factor Loadings	Cronbach Alpha Value	CR	AVE	Discriminant Validity				
Farmers’ subjective well-being		A1	0.854	0.802	0.877	0.704	0.839				
		A2	0.824								
		A3	0.838								
Institutional performance	Ecological livability performance	A4	0.893	0.854	0.886	0.795	0.085 ***	0.839			
		A5	0.890								
	Social Security Performance	A6	0.856	0.698	0.731	0.845	0.07 ***	0.083 ***	0.919		
		A7	0.854								
Government trust	Institutional Trust	A8	0.805	0.848	0.873	0.697	0.098 ***	0.234 ***	0.179 ***	0.835	
		A9	0.883								
		A10	0.814								
	policy trust	A11	0.852	0.698	0.811	0.590	0.023 ***	0.194 ***	0.065 ***	0.156 ***	0.768
		A12	0.741								
		A13	0.703								

Note: ***, **, and * represent the 1%, 5%, and 10% status significance levels, respectively. In discriminant validity, the diagonal line is the square root of the average extraction variance (AVE), and the rest are correlations between latent variables coefficient.

**Table 4:** Model overall fitness evaluation results.

Type	Index	Evaluation Standard	Fitting Results	Whether the Evaluation Criteria Are Met
Absolute fit indicator	CMIN/DF	<3	2.625	Satisfy
	GFI	>0.9	0.978	Satisfy
	AGFI	>0.9	0.963	Satisfy
	RMR	<0.05	0.019	Satisfy
	RMSEA	<0.05	0.041	Satisfy
Relative fit indicator	NFI	>0.9	0.969	Satisfy
	RFI	>0.9	0.956	Satisfy
	IFI	>0.9	0.981	Satisfy
	TLI	>0.9	0.972	Satisfy
	CFI	>0.9	0.981	Satisfy
Streamlined adaptation metrics	PRATIO	>0.5	0.692	Satisfy
	PNFI	>0.5	0.671	Satisfy
	PCFI	>0.5	0.679	Satisfy

**Table 5:** Model Fitting Path Coefficients and Tests.

Action Path		Src.	Std.
Institutional performance→farmers’ subjective well-being	Social insurance performance→farmers’ subjective well-being	0.096 **	0.034
	Ecological livability performance→farmers’ subjective well-being	0.139 ***	0.034
The government trusts the→subjective well-being of farmers	Institutions trust→farmers’ subjective well-being	0.154 ***	0.03
	Policy trusts→farmers’ subjective well-being	−0.015	0.054
Institutional Performance→Government Trust	Social Insurance Performance→Agency Trust	0.171 ***	0.048
	Eco-Living Performance→Institutional Trust	0.356 ***	0.048
	Social Insurance Performance→Policy Trust	0.109 **	0.028
	Ecological Livability Performance→Policy Trust	0.28 ***	0.028

Note: ***, **, * represent the 1%, 5%, and 10% status significance levels, respectively. Std.: standard deviation, and Src.: Standard regression coefficients.

**Table 6:** Results and test of mediation effect.

Action Path		Src.	Std.	Confidence in Terval	
				Lower Limit	Upper Limit
Institutional performance →government trusts →farmers’ subjective well-being	Ecological livability performance →agencies trust →farmers’ subjective well-being	0.006 **	0.004	0.001	0.015
	Social insurance performance →agencies trust →farmers’ subjective well-being	0.018 ***	0.008	0.008	0.034
	The ecological livability performance →policy trusts →farmers’ subjective well-being	−0.003	0.01	−0.021	0.012
	Social insurance performance →policy trusts farmers’ subjective well-being	−0.001	0.004	−0.01	0.004

Note: ***, **, * represent the 1%, 5%, and 10% status significance levels, respectively. Std.: standard deviation and Src.: Standard regression coefficients.

**Table 7:** Moderating effect results and testing.

Action Path		Perception of Welfare Gap between Urban and Rural Areas				Family Economic Status			
		Smaller		Larger		Lower		Higher	
		Src.	Std.	Src.	Std.	Src.	Std.	Src.	Std.
Institutional performance→farmers’ subjective well-being	Ecological livability performance→farmers’ subjective well-being	0.166 ***	0.030	0.097 *	0.041	0.215 ***	0.035	−0.073	0.065
	Social insurance performance→farmers’ subjective well-being	0.117 ***	0.029	0.040	0.041	0.134 **	0.035	0.049	0.062

Note: ***, **, and * represent the 1%, 5%, and 10% status significance levels, respectively. Std.: standard deviation and Src.: Standard regression coefficients.

## Data Availability

The associated data will be provided to the corresponding authors upon request.
